# Use of Behavior Change Techniques in Digital HIV Prevention Programs for Adolescents and Young People: Systematic Review

**DOI:** 10.2196/59519

**Published:** 2025-04-28

**Authors:** Phoenix Kit-han Mo, Luyao Xie, Tsz Ching Lee, Angela Yuen Chun Li

**Affiliations:** 1 Center for Health Behaviours Research JC School of Public Health and Primary Care Chinese University of Hong Kong Hong Kong China (Hong Kong)

**Keywords:** HIV, prevention, adolescent, young adult, behavior change techniques

## Abstract

**Background:**

HIV infections have caused severe public health and economic burdens to the world. Adolescents and young people continue to constitute a large proportion of newly diagnosed HIV cases. Digital health interventions have been increasingly used to prevent the rising HIV epidemic. Behavior change techniques (BCTs) are intervention components designed to modify the underlying processes that regulate behavior. The BCT taxonomy offers a systematic approach to identifying, extracting, and coding these components, providing valuable insights into effective intervention strategies. However, few reviews have comprehensively identified the use of BCTs in digital HIV interventions among adolescents and young people.

**Objective:**

This study aimed to synthesize existing evidence on the commonly used BCTs in effective digital HIV prevention programs targeting adolescents and young people.

**Methods:**

In total, 4 databases (PubMed, Embase, Cochrane Library, and APA PsycINFO) were searched, and studies from January 2008 to November 2024 were screened. Reference lists of relevant review studies were reviewed to identify any additional sources. Eligible randomized controlled trials with 1 of 3 HIV prevention outcomes (ie, HIV knowledge, condom-use self-efficacy, and condom use) were included. Basic study characteristics, intervention strategies, and study results were extracted and compared for data analysis. For the included interventions, BCTs were identified according to the BCT taxonomy proposed by Abraham and Michie in 2008, and the frequencies of BCTs used in these interventions were counted.

**Results:**

Searches yielded 383 studies after duplicates were removed, with 34 (8.9%) publications finally included in this review. The most frequently used BCTs included *prompting intention formation* (34/34, 100%), *providing information about behavior-health link* (33/34, 97%), *providing information on consequences* (33/34, 97%), and *providing instruction* (33/34, 97%). Interventions with significant improvements in HIV knowledge (11/34, 32%) more frequently used BCTs with a provision nature, such as providing information about behavior-health link (11/11, 100%), information on consequences (11/11, 100%), encouragement (10/11, 91%), and instruction (10/11, 91%). Those with significant increases in condom-use self-efficacy (7/34, 20%) used BCTs toward initiating actions, such as prompts for intention formation (7/7, 100%), barrier identification (7/7, 100%), and practice (5/7, 71%). In addition, studies showing significant improvements in condom use (14/34, 41%) included BCTs focused not only on provision and initiation but also on behavioral management and maintenance, such as use follow-up prompts (5/14, 36%), relapse prevention (4/14, 29%), prompt self-monitoring of behavior (3/14, 21%), and prompt review of behavioral goals (3/14, 21%).

**Conclusions:**

This is the first systematic review that examined the use of BCTs in digital HIV prevention interventions for adolescents and young adults. The identified BCTs offer important reference for developing more effective digital interventions, with implications for enhancing their HIV knowledge, condom-use self-efficacy, and condom use in youth.

## Introduction

### HIV Infections Among Adolescents and Young People

HIV continues to be a global public health concern, with around 1.3 million new HIV infections occurring in 2022 worldwide [1]. Until 2022, there were around 39 million people living with HIV worldwide. HIV imposes a tremendous health and economic burden on the world. Since the epidemic began, more than 40 million lives have been lost due to HIV-related illnesses [2]. Lifelong antiretroviral treatments require long-term health care services and medications [3]. A study by Schackman et al [4] estimated that the discounted cost treating one patient with HIV, infected at the age of 35 years, for a lifetime in the United States is approximately US $326,500. By preventing 1 HIV infection, US $229,800 could be saved [4].

Despite a decreasing trend [5], adolescents and young people still occupy a significant proportion of new HIV infection. In 2020, adolescents and young people aged 13 years to 24 years accounted for one-fifth of newly diagnosed cases in the United States [6]. The number of new HIV infections among adolescents and young people from 2010 to 2019 were 170,000 and 460,000, respectively worldwide [5]. Meanwhile, the number of HIV-positive cases of young men who have sex with men (MSM) between the age of 13 years and 24 years has significantly increased from 2015 to 2019, indicating an increasing trend in HIV infection among young MSM in the United States [7]. Significant efforts remain to be made to achieve the global target of ending the HIV epidemic by 2030 [8].

### Behavioral Interventions for HIV Prevention

The disproportionate burden of HIV among adolescents and young people is demonstrated to be primarily driven by HIV risky behaviors, such as unprotected sexual intercourse [9]. It was reported that the percentage of condomless sex among MSM in the United States increased from 46% in 2012 to 70.5% in 2017 [10]. A study suggested that only half (54.3%) of high school students in the United States reported having used condoms during their last sexual intercourse [11]. Hence, improving their condom-use behaviors is important. Previous literature has suggested that HIV knowledge and condom-use self-efficacy were 2 essential cognitive factors influencing condom use [12-15]. Some studies also showed that condom-use self-efficacy was a significant mediator between HIV knowledge and adolescents’ condom use [16]. Nevertheless, the HIV knowledge and condom-use self-efficacy of youth remains suboptimal. It was found that less than half of adolescents possess comprehensive knowledge about HIV prevention [17]. Another study also documented that more than half of university students reported low levels of condom-use self-efficacy [18]. Therefore, to control and prevent HIV among adolescents and young people, interventions to promote their HIV-related knowledge, condom-use self-efficacy, and condom-use behaviors would be particularly important.

### The Internet as a Useful Platform for Health Promotion

In recent decades, increasing use of the internet and smartphones to deliver health interventions has been observed in the literature [19,20]. Numerous advantages of web-based interventions have been identified, such as easy accessibility, availability, and assimilation to participants’ everyday lives. The internet has become the most common platform for young people to search for health-related information [21].

Information on sexual health and sexually transmitted diseases is commonly sought through the internet, especially by ethnic minority groups and minority groups with sexual orientation differences [21,22]. Young MSM are more likely to look for HIV-related information digital compared to the general population [23]. The growing HIV epidemic and the proliferation of the internet use warrants the application of innovative strategies and IT for behavior change interventions. The internet provides an alternative platform for delivering health interventions to the minority population. Accumulated evidence has shown that digital interventions are effective in promoting HIV knowledge and condom use, with varying effect size [24-28].

### Behavior Change Techniques in Health Interventions

One factor contributing to the varying effectiveness of digital HIV prevention can be attributed to the difference in the use of behavior change techniques (BCTs). BCTs are the observable, replicable, and irreducible intervention components that are designed to modify the processes that regulate behavior. In 2008, Abraham and Michie [29] developed a taxonomy of BCTs and defined 26 techniques with respective theoretical support, providing a standardized method of classifying intervention content [29,30]. Theoretical frameworks most frequently identified in the use of BCTs include the information-motivation-behavioral skills model, theory of planned behavior, theory of reasoned action, social cognitive theory, control theory, and operant conditioning [29,31]. Furthermore, as suggested by the Transtheoretical Model, individuals experience stages of precontemplation, contemplation, preparation, action, and maintenance to achieve and sustain a behavioral change [32]. People situated at different stages of change have various needs in driving them onto the next stage, including recognizing the pros and cons of behavior and acquiring the skills to act as well as managing the action to sustain changes. The 26 BCTs categorized in the taxonomy can be classified into various hierarchical levels fulfilling the diverse needs a person requires to produce a behavioral change under the Transtheoretical Model.

Evidence suggests that specific BCTs would be effective in improving a range of health behaviors [33-39]. For example, a systematic review of 19 studies on Type 2 diabetes prevention interventions published in 2020 [37] showed that effective interventions used in general 3.7 to 5.6 times more BCTs than noneffective interventions, and BCTs of social support, goal setting, feedback on behavior, and self-monitoring of outcomes of behavior were identified in >90% of effective interventions. A review and meta-regression published in 2022 [39] identified BCTs used in smartphone app interventions, which found that BCTs of action planning and graded tasks had medium-positive associations with increasing physical activity in people with cardiovascular disease. Another review of interventions that promoted walking and cycling [36] found that prompting self-monitoring of behavior and prompting intention formation were the most frequently coded BCTs in the included studies. Other review studies on interventions in promoting health behavior change also support that those interventions that incorporated more BCTs tended to have larger effects compared to interventions that incorporated fewer techniques [35]. One review specifically targeting mobile health interventions [40] found that techniques of personalization, feedback and monitoring, and associations were most commonly used in mobile health interventions, and prompts and cues were the most common BCTs used in effective trials.

The BCT taxonomy provides a standardized and reliable way to extract and code the information from behavior change interventions and describe their components. It also offers a nomenclature system of intervention techniques that could be applied to health promotion programs aiming to drive behavioral changes [29]. The use of well-defined BCTs allows investigators to evaluate the effectiveness of an intervention more systematically and to conduct accurate replications of efficient interventions. However, past reviews on digital HIV prevention tended to focus on the interventions’ effectiveness, rather than their techniques in leading behavioral changes [25,26,41-43]. There are limited reviews on the BCTs used in digital HIV interventions targeting adolescents and young people [44]. As digital HIV interventions may vary in their content and effectiveness, identifying the most effective BCTs used in different HIV prevention studies is important. This can help to delineate potential mechanisms between components and outcomes and inform the development of more targeted and efficient interventions in the future.

### This Study

This study aimed to identify the commonly used BCTs in effective digital HIV prevention programs targeting adolescent and young people using a systematic review.

## Methods

### Search for Studies

A literature search was conducted in 4 databases, including PubMed, Embase, Cochrane Library, and APA PsycINFO, to identify publications written in English and published from January 2008 (the beginning of the popularization of smartphones) to November 2024 [45]. Search keywords were developed in the following 5 domains: setting, intervention, outcome, population, and study design, using Boolean logic. The search terms in the 4 databases are presented in Multimedia Appendix 1. In addition, bibliographies of existing systematic reviews were also reviewed for a comprehensive data search. No protocol or registration of this review was prepared. This review was performed following the PRISMA (Preferred Reporting Items for Systematic Reviews And Meta-Analyses) statement [46]. The PRISMA checklist is provided in Multimedia Appendix 2.

### Eligibility Criteria

The eligible criteria for this systematic review, developed based on the population, intervention, comparison, outcomes, and study design framework, are provided in Multimedia Appendix 3. Briefly, randomized controlled trials (RCTs) of HIV prevention in adolescents and young people, implementing digital health interventions aiming to improve key outcomes, such as HIV knowledge, condom-use self-efficacy, and condom use, were considered for inclusion.

### Study Selection

Articles were reviewed after a comprehensive search and screening based on the inclusion and exclusion criteria. The screening and selection process was conducted by 2 independent reviewers (TCL and LX) to identify eligible studies. Any disagreements were resolved through discussion and were referred to by a third reviewer (PK-hM) if necessary.

### Data Extraction and Quality Assessment

**Figure 1:**
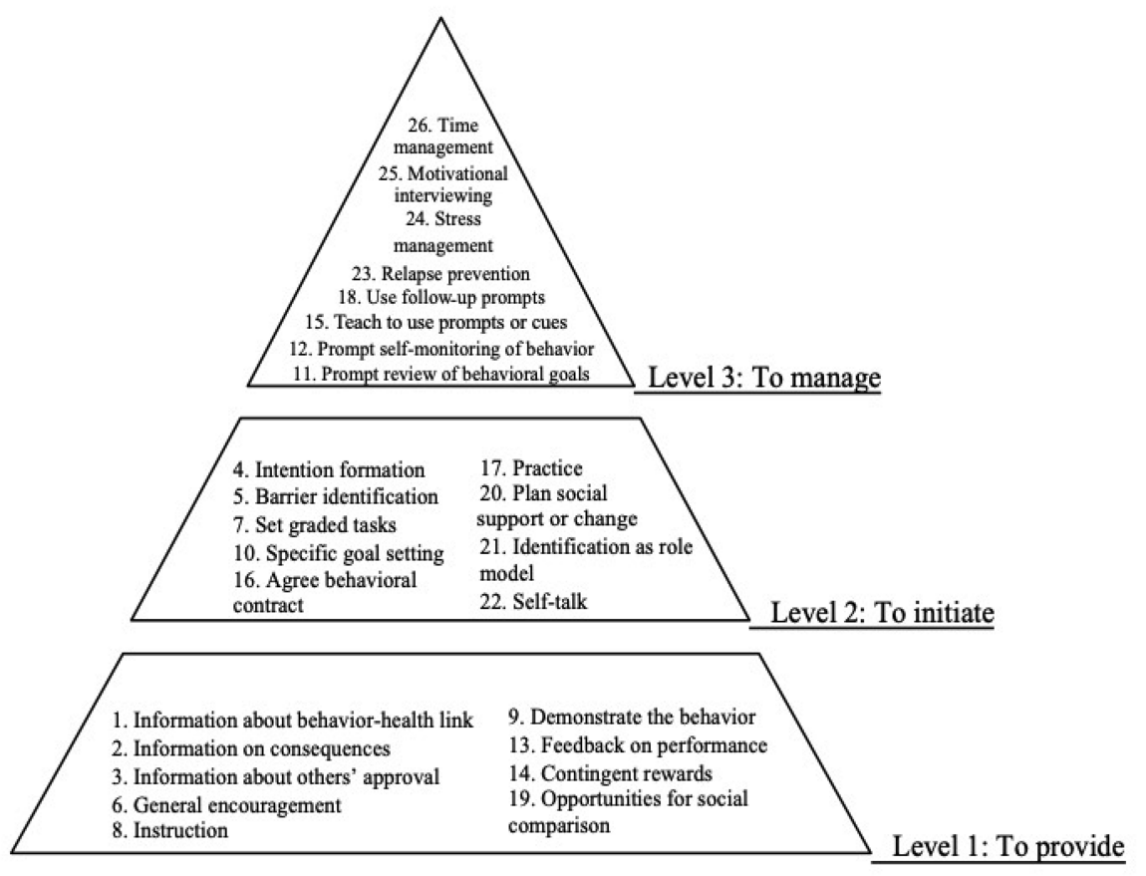
Three hierarchical levels of 26 behavior change techniques developed by Abraham and Michie [29].

For the included studies, data extraction was performed by 2 independent reviewers to collect the following information: studies’ bibliographic information, participant demographics, intervention and control group, models or theoretical frameworks use, intervention intensity, duration, settings, completion rate, and results. The intervention strategies used in each study were reviewed and the BCTs used were identified based on the taxonomy of 26 BCTs developed by Abraham and Michie [29]. The BCTs were further classified into 3 hierarchical levels for individuals with different stages under the transtheoretical model (Figure 1 [29]). In particular, the level 1 BCTs have a provision nature and are used *to provide* information and resources to participants, mainly from a top-down approach. Examples include providing information, providing instruction, and providing general encouragement. Level 2 BCTs aim *to initiate* actions by prompting preparation and planning. Examples include prompt intention formation, barrier identification, specific goal setting, and practice. Level 3 BCTs are used in strategies *to manage* and hence maintain desired health behaviors for a sustainable change during the late action and maintenance stages. Examples are prompt self-monitoring of behaviors, relapse prevention, and time management.

The methodological quality and risks of bias were assessed by the 2 reviewers, using the Joanna Briggs Institute critical appraisal tool for assessment of risk of bias for RCTs [47]. In total, 11 categories, including randomization, concealed allocation, blinding of participants and deliverers, and outcome measurements, were evaluated. An overall percentage score (0% to 100%) was calculated for each study representing the overall study quality. The 2 reviewers scored them independently, and discussion was made with a third investigator in case of any disagreements.

### Data Analysis and Results Interpretation

Basic study characteristics of all RCTs included were summarized in table form. Characteristics included bibliographic information, settings, sample sizes, participant demographics, inclusion criteria, measurements used, and study duration. Results of RCTs were summarized and compared while significant improvements on predefined outcome indicators, as well as intervention completion rate were recorded.

Identified BCTs from intervention of selected RCTs were analyzed and compared based on study outcomes. Application of health models and theories in those studies were noted and matched with relative BCTs. Interventions that were developed or derived from previously published interventions were remarked for potential further investigations. The overall frequencies of BCTs used among all studies collected for this systematic review were counted and summarized. In addition, the frequencies of BCTs used in every significant outcome were counted to explore the commonly used BCTs in effective digital HIV prevention programs targeting adolescent and young audience.

## Results

### Included Studies

**Figure 2:**
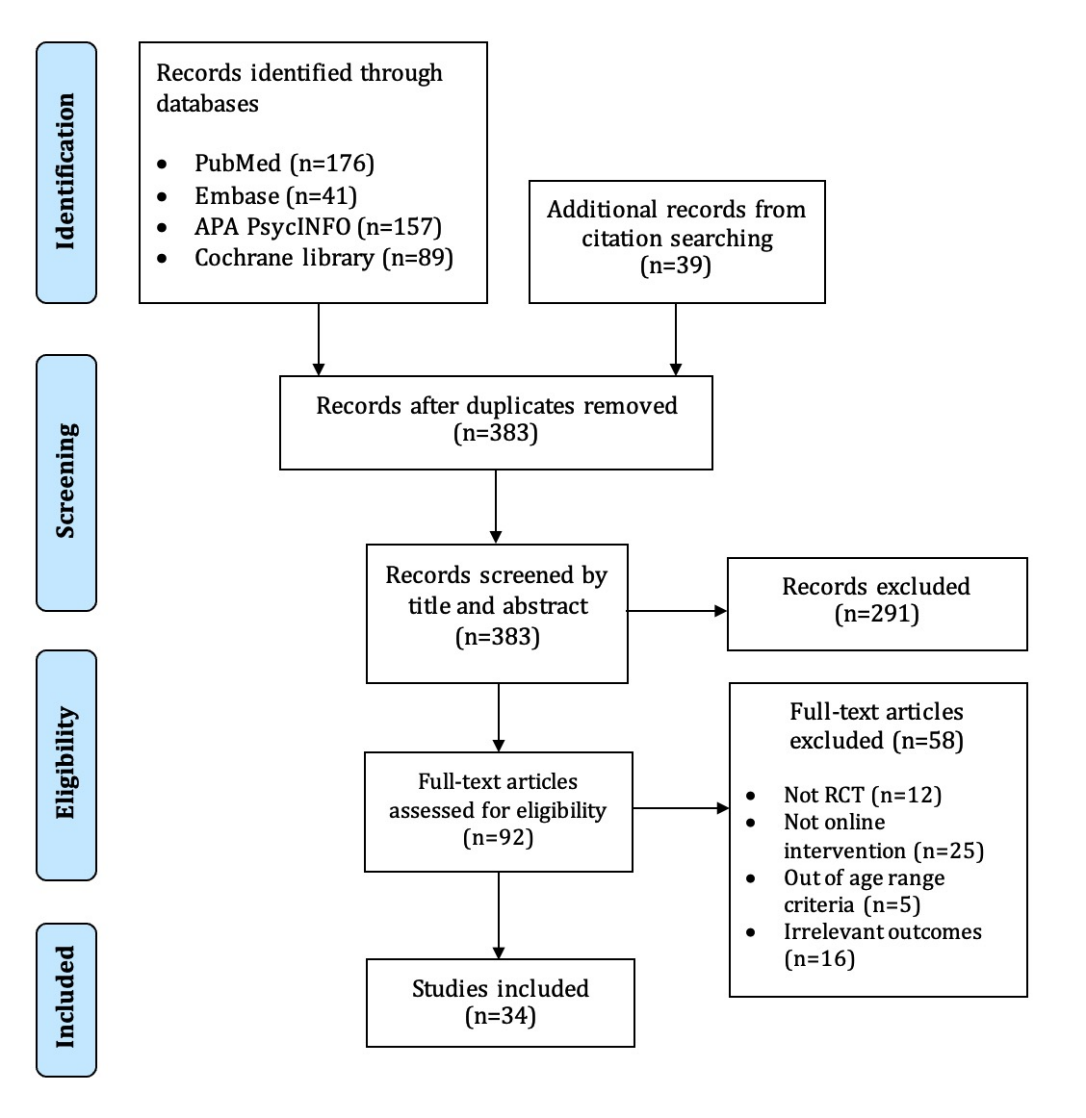
PRISMA (Preferred Reporting Items for Systematic Reviews and Meta-Analyses) Flowchart of the study screening process for this review. RCT: randomized controlled trial.

On the basis of the literature search, 463 records were identified from 4 databases; an additional 39 records were identified through searching for the bibliographies of previous systematic reviews [43,48-50] on digital HIV interventions. Of these records, 119 (25.7%) duplicated records were removed, and the remaining 383 records were screened for eligibility. Following this, an additional 291 (63%) studies were removed after screening by title and abstract, as they did not meet the inclusion criteria. A further full-text review was conducted on the remaining 92 records, and 51 (11%) of them were excluded for the following reasons: non RCT design (n=12, 24%), irrelevant outcomes (n=16, 31%), not digital interventions (eg, in-person interventions, text messaging interventions, or telephone interventions; n=25, 49%), and out of age range (n=5, 10%). Finally, 34 studies were included in this systematic review. The selection process is shown in the PRISMA flowchart in Figure 2 [51].

### Study Characteristics

Table 1 provides an overview of the characteristics of the included studies. Among the 34 studies, 24 (71%) were conducted in the United States, 4 (12%) in African countries (Nigeria [52] and Uganda [53-55]), 5 (15%) in Asian countries (Hong Kong [56], China [57,58], Thailand [59], and Cambodia [60]), and 1 (3%) in Europe (Germany) [61]. Of the 34 studies, 14 (41%) included participants around early or middle adolescence (aged <18 years), and the other 20 (59%) included late adolescents and young people (average age: ≥18 years). In addition, 20 studies (20/34, 59%) targeted at those high-risk populations for HIV infection, involving subgroups such as MSM (n=12, 35%) [56,59,62-71], sexual minority men (N=1) [57], transgender women (n=1, 3%) [59], Black people (n=3, 9%) [67,68,70], low-income [72,73] or homeless individuals [74], refugees (n=1, 3%) [55], female entertainment workers (n=1, 3%) [60] and people with substance use [75,76]. The sample size in the 34 included studies ranged from 40 [62] to 1578 [77].

**Table 1:** Sample characteristics of included studies (N=34).

Author (year)	Country	Sample size, n	Recruitment	Participants			
				Targeted population (age range, if mentioned) and their mean age	Race and ethnicity	Male (%)	If were considered a high-risk population
Anand et al [59], (2020)	Thailand	76 (IG^a^:37, CG^b^:39)	Websites and testing clinics	Late adolescents and young people (aged ≥18 years), median 28 years	Thai nationals	75 (98.7; MSM^c^ and 1 transgender women)	Yes (MSM or transgender women)
Bauermeister et al [70], (2019)	United States	180 (IG:120, CG:60)	Websites and social media	Late adolescents and young people (aged 18 to24 years), mean 21.7 years	White, Black, Asian, Middle Eastern, Native American, and mixed race	100	Yes (Black and MSM)
Brody et al [60], (2022)	Cambodia	1118 (IG: 435; CG: 683)	Entertainment venues	Female entertainment workers (aged 18 to 30 years), mean 24.6 years	Cambodian	0	Yes (female entertainment workers)
Bull et al [78], (2009)	United States	1565 (IG:799, CG: 766)	Websites	Late adolescents and young adulthood (aged 18-24 years), mean22 years	White, Asian, Black, Hispanic, multiracial, and others	42	No
Bull et al [77], (2012)	United States	1578 (IG:942, CG:636)	Websites and newspapers	Late adolescents and young adulthood (aged 15 to 25 years), mean 20 years	Hispanic, African American, Native American, Asian, Hawaiian, White, and others	Unknown	No
Christensen et al [69], (2013)	United States	935	Websites	Late adolescents and young adulthood (aged 18 to 24 years), mean: 21 years	Black, Latino, and White	100	Yes (MSM)
Cordova et al [79], (2020)	United States	50 (IG:25, CG:25)	Health clinics	Middle and late adolescents (aged 13 to 21 years), mean 19 years	White, Black, Native American, and others	18	No
Ezegbe et al [52], (2018)	Nigeria	80 (IG:40, CG: 40)	Schools	Middle adolescents (junior secondary school students), mean 14.78 years	Not mentioned	52.5	No
Fiellin et al [80], (2017)	United States	333 (IG: 166, CG: 167)	Schools	Early adolescents (aged 11 to 14 years), mean 12.9 years	White, Black, and others	53	No
Hightow-Weidman et al [68], (2012)	United States	50 (IG: 25, CG: 25)	Websites and community flyers	Late adolescents and young people (aged 18 to 30 years), mean 23.7 years	Black	100	Yes (Black and MSM)
Hightow-Weidman et al [67], (2019)	United States	474 (IG: 238, CG:236)	Websites and local flyers	Late adolescents and young people (aged 18 to 30 years), mean 24.3 years	Black	100	Yes (Black and MSM)
Jones et al [81], (2013)	United States	295 (IG:149, CG:146)	Testing clinics and community centers	Late adolescents and young people (aged 18to 29 years) mean 22 years	African American, Latino, and Caribbean	0	No
Klein and Card [82], (2011)	United States	178 (IG: 91, CG:87)	Websites and community flyers	Middle and late adolescents (aged 14 to 19 years), mean 15.8 years	Black	0	No
Lau et al [56], (2016)	Hong Kong SAR	402 (IG1: 133, IG2:133, CG:136)	Websites and gay venues	Late adolescents and young people (aged ≥18 years), mean or median not mentioned	Asian	100	Yes (MSM)
Levy et al [61], (2021)	Germany	149 (IG:72, CG:77)	Universities	Late adolescents and young people (university students, aged 18 to 30 years), mean 21.48 years	Not mentioned	15.4	No
Logie et al [55], (2023)	Uganda	450 (IG1: 152; IG2: 157; CG: 141)	Refugee settlements	Youth (aged 16 to 24 years), mean 20 years	Not mentioned	50.7	Yes (refugee)
Marsch et al [75], (2011)	United States	56 (IG:28, CG:28)	Clinics	Early and middle adolescents (aged 12 to 18), mean: 16 years	African American, White, and others	70	Yes (substance abuse)
Marsch et al [76], (2015)	United States	141 (IG:69, CG:72)	Clinics	Early and middle adolescents (aged 12 to 18 years), mean: 16 years	Black, White, multiracial, and others	77	Yes (substance abuse)
McCrimmon et al [83], (2024)	United States	457 (IG: 233, CG: 224)	High School	9th and 11th graders Adolescents (no age restriction reported), mean 15.1 years	White, Black, Hispanic, and others	40	No
Mustanski et al [66], (2013)	United States	102 (IG:50, CG:52)	Testing clinics	Late adolescents and young adulthood (aged 18 to 24 years), mean 21 years	Latino, White, African American, and others	100	Yes (MSM)
Mustanski et al [65], (2018)	United States	901 (IG 445, CG:456)	Testing clinics	Late adolescents and young people (aged 18 to 29 years)	White, Black, Latino, and others	100	Yes (MSM)
Nelson et al [64], (2022)	United States	154 (IG:77, CG:77)	Websites and social media	Middle adolescents (age 14-17), mean: 16	White, Black, Latino, mixed race, and others	100	Yes (MSM)
Newcomb et al [71], (2023)	United States	400 (IG: 200, CG: 200)	Social media and dating or hookup apps	Young men (aged 18 to 29 years) mean 28.3 years	White, Hispanic or Latinx, Black, Asian, Native Hawaiian or Pacific Islander, Native American or Alaskan Native, multiracial, and others	100	Yes (MSM)
Peskin et al [73], (2015)	United States	1374 (IG:768, CG:606)	Schools	Middle adolescents (Eighth grade students), mean 14.3 years	African American, Hispanic, and others	41	Yes (Most students were economically disadvantaged)
Santa Maria et al [74], (2021)	United States	97 (IG: 48, CG:49)	Community centers	Late adolescents and young adulthood (aged 18 to 25 years), mean: 21.2 years	White, Black, Latino, others, and multiracial	57	Yes (homeless)
Schnall et al [63], (2022)	United States	763 (IG: 382, CG:381)	Websites and community centers	Middle adolescents (aged 13 to 18 years), mean 16.2 years	Black, Latino, and White	100	Yes (same-sex attracted adolescent males)
Swendeman et al [84], (2024)	United States	1482 (IG1: 266, IG2: 279, IG3: 295, CG: 642)	Community centers and clinics	Adolescents (aged 12 to 24 years), mean: 21.1 years	Black or African American, Latinx or Hispanic, White (non-Hispanic), Asian or Pacific Islander, and Other or mixed background	93	No
Widman et al [72], (2018)	United States	222 (IG:107, CG:115)	Schools	Middle adolescents (10th grade students), mean: 15.2 years	White, black, and Hispanic	0	Yes (low-income)
Widman et al [85], (2020)	United States	226 (IG： 113, CG:113)	Schools	Middle adolescents (10th and 11th grade students), mean 16.3 years	White, Black, Hispanic, and others	42	No
Wray et al [62], (2019)	United States	40 (IG:20, CG: 20)	Gay-oriented smartphone dating apps	Late adolescents and young people (aged 18to 30 years), mean 28 years	White, black, Asian, and multiracial	100	Yes (MSM with heavy drinking problems)
Ybarra et al [53], (2013)	Uganda	366 (IG: 183, CG: 183)	Schools	Middle adolescents (Secondary students, aged >12 years), mean 16.1 years	Not mentioned	84	No
Ybarra et al [54], (2015)	Uganda	366 (IG: 183, CG: 183)	Schools	Middle adolescents (Secondary students, aged >12 years), mean: 16.1 years	Not mentioned	84	No
Yi et al [57], (2024)	China	120 (IG: 60, CG: 60)	Social media for sexual minority men and community centers	Young men (aged 16 to 30 years), mean 23.2 years	Han, Hui, Miao, and Tujia	100	Yes (Sexual minority men)
Zhang et al [58], (2024)	China	247 (IG: 125, CG: 122)	Factories	Men (>18 years), mean 36.3 years	Han, minority group	100	No

^a^IG: intervention group.

^b^CG: control group.

^c^MSM: men who have sex with men.

Regarding recruitment methods of the included RCTs, 8 (23%) recruited participants from educational institutions (n=7, 12% from secondary schools [52-54,72,73,80,83,85] and n=1, 3% from a university [61]), 7 (12%) recruited through a combination of digital advertisements and community-based organizations [56,57,59,63,67,68,77,82], 7 (12%) recruited from community centers or treatment clinics [65,66,74-76,79,81,84], 1 (3%) recruited from factories [58], 1 (3%) recruited from refugee settlements [64], 1 (3%) recruited from entertainment venues [60], and 5 (15%) recruited entirely digital via websites, social media, or dating apps [62,64,69-71,78]. Most of the studies focused on participants who were HIV-negative (Table 1).

### Intervention Characteristics

An overview of the characteristics of interventions included is listed in Tables 2 and 3*.* Most of the studies (29/34, 85%) were 2-arm intervention design with 1 experimental group and 1 control group. In total, 4 studies [53-56] had 2 experimental groups together with 1 control group. Only 1 study [84] had 3 experiential groups together with 1 control group. In addition, 6 studies [56,58,62,72,83,85] were one-off interventions; the remaining 28 studies had multiple sessions, and their intervention duration ranged from 2 weeks [76] to 24 months [84].

**Table 2:** Intervention conditions and effectiveness of included studies (N=34).

Author (year)	Modality type	On-site session	Time of follow-up (from baseline)	Completion rate after intervention (%)
Anand et al [59], (2020)	Website	No	6 mo and 12 mo	88.2
Bauerm-eister et al [70], (2019)	Mobile app	No	1 mo, 2 mo, and 3 mo	79.4
Brody et al [60], (2022)	SMS text messaging	No	6 mo and 12 mo	34.7
Bull et al [78], (2009)	Website	No	2 mo (internet sample) or 3 mo (clinic sample)	Not mentioned
Bull et al [77], (2012)	Social media	No	After intervention (8 wk from baseline), 6 mo	69
Christensen et al [69], (2013)	Video game	No	After intervention and 3-mo after intervention	98.5
Cordova et al [79], (2020)	Mobile app	Yes	After intervention, 30 d	98
Ezegbe et al [52], (2018)	Social media	No	8 wk and follow-up after intervention	100
Fiellin et al [80], (2017)	Video game	Yes	6 wk, 3 mo, 6 mo, and 12 mo	83
Hightow-Weidman et al [68], (2012)	Website	No	1 and 3 mo	90
Hightow-Weidman et al [67], (2019)	Website	No	3, 6, and 12 mo	85.2
Jones et al [81], (2013)	Website	No	3 and 6 mo	80.7
Klein and Card [82], (2011)	Multimedia software	Yes	3 mo	91
Lau et al [56], (2016)	Website	No	After intervention, 1 mo, and 3 mo	77.4
Levy et al [61], (2021)	Website	No	1 mo	69.1
Logie et al [55], (2023)	SMS	No	8 mo, 12 mo, and 16 mo	71.1
Marsch et al [75], (2011)	Multimedia software	Yes	After intervention, 1-mo after test, and 3-mo after test	93
Marsch et al [76], (2015)	Multimedia software	Yes	After intervention (within 2 wk)	100
McCrimmon et al [83], (2024)	Website	Yes	After intervention	100
Mustanski et al [66], (2013)	Website	No	After intervention, 6, and 12 wk	88.2
Mustanski et al [65], (2018)	Website	No	3, 6, and 12 mo	81.2
Nelson et al [64], (2022)	Website	No	3 wk and 15 wk	89
Newcomb et al [71], (2023)	Videoconference	No	3 mo, 6 mo, 9 mo, and 12 mo	58.3
Peskin et al [73], (2015)	Video game	Yes	1 y	89.2
Santa Maria et al [74], (2021)	Mobile app	No	6 wk	Not mentioned
Schnall et al [63], (2022)	Mobile app	No	3 mo, 6, and 9 mo	81.7
Swendeman et al [84], (2024)	SMS, social media, and videoconference	No	4 mo, 8 mo, 12 mo, 16 mo, 20 mo, and 24 mo	42.2
Widman et al [72], (2018)	Website	No	After intervention, 4 mo	99.5
Widman et al [85], (2020)	Website	No	After intervention	91.6
Wray et al [62], (2019)	Mobile app	Yes	1, 2, and 3 mo	97.5
Ybarra et al [53], (2013)	Website	Yes	3 and 6 mo	94.8
Ybarra et al [54], (2015)	Website	Yes	3 and 6 mo	94.8
Yi et al [57], (2024)	Website	No	4 mo and 8 mo	95
Zhang et al [58], (2024)	Videos	No	6 mo and 12 mo	89.1

**Table 3:** Conditions and outcomes of included interventions.

Author (year)	Number of groups	Intervention condition 1	Intervention condition 2	Control condition	Outcomes measured	Results
Anand et al [59], (2020)	2	An digital video-based discussion and interaction platform, Vialogues, with monthly HIV education sessions. The website was integrated with counseling and testing support services, as well as video-uploading and messaging functions for counselors and participants to discuss.	N/A^a^	Private clinic-based HIV counseling and testing.	HIV knowledge, percentage of condom use for anal intercourse, condom-use self-efficacy	Unchanged HIV knowledge level at baseline and after intervention in both arms; significant increase in percentage of condom use in the intervention group; no significant changes in condom-use self-efficacy among intervention group participants.
Bauerm-eister et al [70], (2019)	2	A mobile app, myDEX, equipped with 6 sessions on sexual health information and skills tailored for YMSM^b^. Each session consisted of a core message, a deep discussion regarding the topics, as well as an interactive activity. Features like a diary, role-playing, quizzes, and practice opportunities were included.	N/A	An Information-only static site with HIV prevention contents split into 6 sessions.	Condomless anal sex events and condom-use self-efficacy	Intervention participants were less likely to engage in condomless anal sex than participants from the control group; control participants were more likely to forego condoms than participants from the intervention group
Brody et al [60], (2022)	2	Participants could choose to receive text or voices messages. 180 messages comprising of 10 health themes will be sent, including gender-based violence, cervical cancer, vaginal health, contraception, hygiene, pregnancy termination, general health information, alcohol use, HIV and STI^c^ transmission and prevention, pregnancy and miscarriage. A message was sent 2 times for 10 wk, and the message from each topic area was repeated every 10 wk for 60 wk. An option to be contacted by outreach workers for free was provided after each message.	N/A	Existing standard care.	HIV test, STI testing when symptoms arise, contraceptive usage, and condom use with nonpaying and paying partner	No significant differences in HIV-preventive behaviors, including HIV or STI testing, contraceptive usage and condom use with nonpaying and paying partner.
Bull et al [78], (2009)	2	An interactive website, Keep It Real, was developed. It contained 5 modules with 60-90 s long role model stories focusing on condom use and HIV risks. Quizzes were included to test understanding. A booster session at 1-mo follow-up showed the same messages again.	N/A	A computer kiosk at a clinical setting with text-based information on condom use and HIV risks. Quizzes were included to test understanding. A booster session at 1-mo follow-up showed the same messages again.	Proportion of protected sex acts by condoms and condom-use self-efficacy	No significant effects on protected sex acts and condom-use self-efficacy.
Bull et al [77], (2012)	2	A Facebook page, “Just/US,” was developed and ran for 2 mo. Eight topics focusing on communication skills and condom-use efficacy were delivered through daily contents in different formats, eg, videos, quizzes, games, and discussions.	N/A	A Facebook page called “18-24 News” showing interesting news tailored for people aged 18-24 from 6 PM to midnight. Sexual health contents were avoided.	Condom use at last sex and proportion of protected sex acts by condoms	Condom use at last sex remained stable in intervention group but decreased in the control group.; proportion of protected sex acts remained stable in intervention group but significantly decreased in the control group.
Christensen et al [69], (2013)	2	A simulation video game, SOLVE, was used to simulate common obstacles encountered by YMSM at different scenarios. Depending on participants’ decisions, different choice points would be awarded. Sexuality affirmations were included to reduce shame for MSM^d^.	N/A	No intervention.	Number of unprotected anal intercourse UAI^e^	Significant indirect decrease in number of UAI caused by shame reduction in the intervention group
Cordova et al [79], (2020)	2	In addition to the clinic’s usual services, a mobile app, Storytelling 4 Empowerment, was developed to deliver HIV, tobacco, and substance use knowledge through 3 modules. A clinician version with participants’ history and risk assessments was also developed to facilitate communication during in-person counseling sessions.	N/A	The clinic’s usual services on sexual health as well as a printed copy of Storytelling 4 Empowerment tobacco module contents.	Sexual risk self-efficacy, sexual risk prevention knowledge, sexual risk behaviors	Small insignificant effect sizes in sexual risk self-efficacy change scores within the intervention group.; larger gains of sexual risk prevention knowledge, including HIV, in intervention group compared to the control group; greater, but insignificant reduction in condomless sex among intervention participants than control participants.
Ezegbe et al [52], (2018)	2	An 8-wk long, twice a week, digital storytelling intervention. The REDStory intervention contained digital sessions with videos from social media platforms, as well as offline therapist discussion sessions with video narration activities.	N/A	No intervention	HIV knowledge	Significant improvement in HIV knowledge among schoolchildren from intervention group than control group
Fiellin et al [80], (2017)	2	A 2D role-playing adventure game, PlayForward, with interactive features where players can make their own decisions in different social contexts to learn about HIV prevention; 2 one-h sessions per week for 6 wk.	N/A	Twelve irrelevant video games, eg, Angry Birds, with the same number and length of sessions as the experiment group.	Sexual health knowledge	Significant increase in sexual health knowledge in intervention group compared to control group
Hightow-Weidman et al [68], (2012)	2	The website, HealthMpowerment.org, contained 7 main sessions with interactive features, including resources, quizzes of different levels, personalized health, and sex journals. Included. four 30-min weekly sessions	NA	A list of 5 Web sites providing general HIV information.	Number of protected sex acts, condom-use self-efficacy, and HIV knowledge	Significant increase in self-reported condom use among participants from both groups at 1-mo follow-up; no significant difference in condom-use self-efficacy and HIV knowledge between the intervention and control group.
Hightow-Weidman et al [67], (2019)	2	Website, HealthMpowerment.org, was revised with a knowledge library on HIV prevention with interactive features, including discussion forums, personal space for uploading information and digital physician consultation.	NA	Website with a smaller-scale knowledge library.	Number of acts of CAI^f^	Significant decline in rate of CAI in the intervention group compared to the control group.
Jones et al [81], (2013)	2	A 12-wk soap opera video series, *Love, Sex, and Choices* was produced and streamed to smartphones. Each episode was 15-20 min long with contents describing different high-risk situations. Principles of HIV risk reduction were modeled by main characters.	N/A	A 12-wk text messaging intervention containing HIV risk reduction written messages delivered by smartphones.	Percentage unprotected vaginal and anal sex acts	Significant decline in unprotected sex acts in both the intervention and control group; intervention group had a lower percentage of unprotected sex acts compared to the control group at follow-up, given they were the same at baseline.
Klein and Card [82], (2011)	2	A computer-delivered multimedia software developed from an in-person intervention SiHLE. It contained 2 1-h sessions, centering on HIV prevention and sexual decision-making skills delivered through multimedia channels, including games, quizzes, and videos. All sessions were narrated by teenage female health educators.	N/A	A general health education session with 2 laptop-delivered videos about healthy diet and nutritious eating. The 2 videos were 65 min long in total.	HIV knowledge, condom-use self-efficacy, and percentage of condom protected vaginal intercourse acts	Significant improvement in HIV knowledge in both intervention and control group, with higher improvement among intervention group; significant increase in condom-use self-efficacy among nonsexually active participants in the intervention group; significant increase in percentage condom use among intervention participants, compared to no change in condom use in the control group.
Lau et al [56], (2016)	3	A website with videos based on STD^g^-related cognitions. Contents of the videos included HIV and syphilis information as well as means of prevention.	In addition to videos based on STD-related cognitions from intervention 1, a fear-arousing video based on STD-related emotions was added in intervention 2, highlighting consequences of social loss due to STDs.	A website with text HIV-related facts and information.	Number of UAI	No significant association between 3 treatment groups and prevalence of UAI was discovered; significant within-group reduction in UAI in all 3 groups; significant reduction in UAI with casual sex partners in intervention group 2 with fear appeal.
Levy et al [61], (2021)	2	Psychological inoculation was used to design an interactive website with HIV knowledge as well as 10 challenging sentences regarding condom use barriers and social pressure. Participants had to refute relative statements. Exaggerated versions would be provided for weak refutation.	N/A	A static website with HIV knowledge and 10 additional true or false questions on condom use to control for the experimental group.	Frequency of condom use	Frequencies of condom use increased in control group but not experiment group.
Logie et al [55], (2023)	3	Weekly SMS will be sent to ask how they are doing and have 2 choices of response, “fine” or “not fine.” Participants who responded “not fine” or did not respond will be followed-up by a peer navigator within 2 d and a week, respectively [86].	HIV self-testing kit, along with instruction, condoms, lubricant, information pamphlet, and referral information for confirmatory testing	Information about HIV testing and services at local clinics and a leaflet on HIV-preventive strategies [86].	HIV testing frequency, knowledge of HIV status, condom-use self-efficacy, and consistent condom use [86]	Significant increase in HIV testing, HIV status in knowledge in both intervention groups comparing to control group; significant improvement in condom usage self-efficacy comparing intervention groups and the control group; not significant difference in consistent condom use comparing the intervention groups and control group.
Marsch et al [75], (2011)	2	On top of the traditional intervention (the same as the control measures), a self-directed web-based program which contained 19 modules on HIV risk reduction and relative risks brought by drug abuse would be added. The system provided customized selection of modules based on participants’ risk assessment survey results.	N/A	A 1-h individual or small group educator-delivered sessions by trained HIV prevention specialists on basic HIV-related information as well as drug-related risks. In addition, a 15 min long video was played.	HIV prevention knowledge, condom-use self-efficacy	Significant increase in HIV prevention knowledge in both groups, while participants from intervention group showed larger increases compared to that of control group; significant increase in skills to correctly use condoms in both groups without intergroup differences.
Marsch et al [76], (2015)	2	A self-directed web-based program, the Therapeutic Education System, which contained 19 modules on HIV risk reduction and relative risks brought by drug abuse. The system provided customized selection of modules based on participants’ risk assessment survey results.	N/A	Two 1-h individual or small group educator-delivered sessions by trained HIV prevention specialists on basic HIV-related information as well as drug-related risks. In addition, a 20 min long video was played.	HIV knowledge and condom-use self-efficacy	Increase in HIV knowledge in both groups without significant intergroup differences; increase in condom-use self-efficacy in both groups without significant intergroup differences.
McCrimmon et al [83], (2024)	2	A self-directed web-based program adapted Health Education and Relationship Training, which included 6 modules on safe sex motivation, sexual communication skills, HIV and STI knowledge, safer sexual self-efficacy, and sexual norms and attitudes. Interactive activities include games, animated characters and quizzes [87].	N/A	A 45-min attention matched web-based program that targets growth mindsets was delivered [87].	Condom use intention, attitude and norm, HIV and STI knowledge, and safe sex communication self-efficacy	Higher improvement in attitude toward condom and HIV and STI knowledge in intervention group compared to control group; no significant difference in condom use intention and norm; safe sex communication self-efficacy
Mustanski et al [66], (2013)	2	Three digital sessions with 7 interactive modules in the Keep It Up! Intervention website (around 2 h in total). Different contents, including videos, animations, and games, were implemented. Contents were tailored to YMSM. A 6-wk booster session was applied.	N/A	Same number of modules and sessions as the intervention group with didactic noninteractive texts and images on HIV-related information. No tailored contents for YMSM.	Number of unprotected anal sex acts and HIV knowledge	A small decrease in rate of unprotected anal sex acts in intervention group, but an increase in the control group; a large increase in HIV knowledge in both groups, with no significant difference between groups.
Mustanski et al [65], (2018)	2	Three digital sessions with 7 interactive modules in the Keep It Up! Intervention website (around 1 h in total). Different contents, including videos, animations, and games, were implemented. Contents were tailored to YMSM. 3- and 6-mo booster sessions were applied.	N/A	Same number of modules and sessions as the intervention group with static texts and images on HIV-related information. No tailored contents for YMSM.	Incident STI, reporting number of casual CAS^h^ acts and CAS partners	Significant reduction in STI and reported CAS for intervention group versus control group; no difference between intervention and control groups regarding incident HIV.
Nelson et al [64], (2022)	2	Interactive website with 9 modules focusing on 4 main topics, including male anatomy, HIV or STI-related knowledge, general sexual health information, and pornography truths. The website included adventure games, questions and answers videos, and illustrations. Participants needed to unlock the first 3 modules before given the access to the remaining 6 modules.	N/A	Website of Centers for Disease Control and Prevention, as well as Web sites of national HIV and STI testing resources.	HIV knowledge and condom knowledge	Insignificant differences of HIV knowledge scores among intervention and control group; no difference on condom knowledge scores among intervention and control group.
Newcomb et al [71], (2023)	2	Five sessions regarding sexual health, HIV risk and relationship functioning were conducted. Three videos on these topics were sent to participants each time, then 3 videoconference group sessions were implemented to build skill. Two individual coaching video conference for each coupon were delivered for skill implementation.	N/A	A single 90-min session. HIV-negative or unknown couple: Testing Together protocol (HIV testing and education). HIV-Positive: Medication and Risk Reduction Counseling (explore motivators and barriers to antiretroviral adherence and plan for it). Serodiscordant: receive both of the above.	STI, CAS, HIV testing, and PrEP^i^ use	Significantly lower STI positive cases, CAS frequency in intervention group; no difference on HIV testing and PrEP use among intervention and control group.
Peskin et al [73], (2015)	2	An interactive computer-based video game, IYG-Tech, with thirteen 45 min long modules on sexual health. Animated scenarios, videos, quizzes, fact sheets, role-playing and discussion platforms were built-in activities aiming to equip students with necessary sexual health life-skills.	N/A	Standard textbook health education.	Number of condomless sex, knowledge of STIs, and condom-use self-efficacy	No significant differences in the number of condomless sex among the 2 groups; intervention students had greater knowledge about STIs and higher condom-use self-efficacy than control students in the 1-y follow-up.
Santa Maria et al [74], (2021)	2	A mobile app, MY-RID, was delivered to homeless young adults. Participants firstly set a goal of behavioral change for HIV prevention, then answered daily assessment questions (EMA^k^) followed by tailored messages regarding their specific goal and real-time risk predictors.	N/A	The same mobile app and assessment items were applied, but the focus was on general health behaviors. General messages, instead of tailored ones, would be received.	Frequency of condomless sex acts	No significant intervention effect was observed on frequency of condomless sex acts.
Schnall et al [63], (2022)	2	A mobile app, MyPEEPS Mobile, was developed based on a group-based intervention. It focused on the stories of 4 YMSM (peeps) and aimed to build knowledge, self-awareness, and self-efficacy in sexual risk reduction through 21 mobile activities, completed throughout a 3-mo period.	N/A	Delayed intervention receiving MyPEEPS mobile at 9-mo follow-up after data collection.	Number of condomless sex acts	Significant reduction in condomless anal sex acts among intervention group participants, compared to the control group participants; most pronounced and long-lasting effect on Black participants, compared to participants of other races.
Swendeman et al [84], (2024)	4	IG^j^1: coaching and peer support and SMS text messaging; coaching: weekly telehealth strength-based coaching, 30-min for first 2 mo then 5-20 min following, covering assessment, linkage to services, goal setting, problem solving, and cognitive and behavioral skills training; peer Support: up to 16 wk to discuss and support on a moderated digital private forum; seed discussion topics were posted twice a week [88]; SMS text messaging: Daily SMS sent for 24 mo, covering sexual health, physical and mental health, substance use, and medication reminders if applicable, and a weekly self-monitoring survey on HIV or STI symptoms, risky sexual behaviors, and medication adherence.	IG2: coaching and SMS text messaging; IG3: peer support and SMS text messaging	SMS text messaging only.	PrEP use and adherence, consistent condom use, and PEP prescription and adherence	All intervention groups had higher increase in PrEP use compared to the control group; intervention group1 had sustained an increase in PrEP use compared to all other groups; no significant differences between PrEP adherence, PrEP prescription and adherence.
Widman et al [72], (2018)	2	A 45 min long digital intervention, HEART for Girls, with 5 modules targeting motivations, HIV knowledge, social norms, safer sex self-efficacy, and sexual communication skills. Sexual assertiveness skills were emphasized throughout all modules.	NA	A 45 min long attention-matching digital intervention, Growing Minds, with 5 modules on academic and social growth mindsets.	HIV knowledge, condom-use self-efficacy, and condom use at last sexual intercourse	Significant increase in HIV knowledge in the intervention group compared to the control group; significant increase in condom-use self-efficacy in the intervention group compared to the control group; better, but insignificant condom use among participants in the intervention group compared to the control group.
Widman et al [85], (2020)	2	A 45 min long digital intervention, HEART for Teens, with 5 modules targeting motivations, HIV knowledge, social norms, safer sex self-efficacy, and sexual communication skills. Sexual assertiveness skills were emphasized throughout all modules. More male characters were added compared to HEART for Girls.	N/A	A 45 min long attention-matching digital intervention, Growing Minds, with 5 modules on academic and social growth mindsets.	HIV knowledge and condom-use self-efficacy	Higher HIV knowledge and condom-use self-efficacy in intervention group compared to control group; similar intervention effects across 2 boys and girls.
Wray et al [62], (2019)	2	In addition to the standard posttest counseling services, a mobile app, Game Plan, was provided after HIV testing. Reflective exercises to prompt self-talk and goal setting functions were incorporated into the app to motivate behavioral change. This is a one-off intervention with follow-up surveys for 3 mo.	N/A	Standard post-HIV testing counseling with referral services.	Number of CAS events	Insignificant effect on number of CAS events in participants from both the intervention and control group.
Ybarra et al [53], (2013)	3	The CyberSenga website had 5 1-h intervention modules focusing on information about HIV and healthy sexual relationship, as well as skills needed for decision-making and condom use. Four versions of materials were developed tailoring different needs according to participants’ genders and sexual experiences.	Same as the first intervention group but with a booster module at 4 mo after intervention.	Treatment as usual: no extra interventions other than HIV programs currently being provided at schools.	Reporting number of unprotected sex acts in the past 3 mo	No significant differences at the rates of unprotected sex in intervention and control groups in the past 3 mo.
Ybarra et al [54], (2015)	3	The CyberSenga website had 5 1-h intervention modules focusing on information about HIV and healthy sexual relationship, as well as skills needed for decision-making and condom use. Four versions of materials were developed tailoring different needs according to participants’ genders and sexual experiences.	Same as the first intervention group but with a booster module at 4 mo after intervention.	Treatment as usual: no extra interventions other than HIV programs currently being provided at schools.	HIV prevention-related information	Intervention group participants answered greater percentages of questions correctly, compared with control group participants; improvement of HIV-related knowledge was the greatest among intervention participants with the booster module.
Yi et al [57], (2024)	2	LGBTQ^k^-affirmative cognitive behavioral therapy: 10 modules covering goal setting, LGBTQ-related stress and its reactions, automatic thoughts, emotion avoidance, emotion-driven behaviors, and behavioral skill training and experiment. Each module contained videos, 4-5 pages of psychoeducation, exercises and homework.	N/A	Weekly self-monitoring survey for 10 wk.	HIV-transmission-risk behaviors, HIV or syphilis result, condom self-efficacy, perceived condom use benefits, and mental and behavioral health	No significant differences in HIV-transmission-risk behaviors and social cognitive mechanisms between intervention group and control group; no positive HIV or syphilis cases reported; intervention group showed greater improvements in depression and anxiety in follow-ups.
Zhang et al [58], (2024)	2	Different web-based videos based on participants’ behavioral assessment results: (1) video for those at lower risk: promote capacity to refuse peers’ invite to have sex with female sex workers or nonregular female sex partners. Content includes high-risk nature, sex partner may be asymptomatic, severe interpersonal consequences, transmit to HIV or STI to stable partners; (2) video for people at high risk to promote their HIV testing: the benefits of taking up HIV testing and the procedures of free HIV testing and counseling at local Centers for Disease Control	N/A	Basic HIV-related knowledge.	Sexual intercourse with female sex partners and sex workers, respectively, condomless sex with female sex partner and sex worker, respectively, and uptake of HIV testing	Significant difference was observed in 6-mo follow-up in lower frequency of sexual intercourse with nonregular female sex partners of intervention group, compared with the control group; no significant differences in all other outcomes or time points.

^a^N/A: not applicable.

^b^YMSM: young men who have sex with men.

^c^STI: sexually transmitted infection.

^d^MSM: men who have sex with men.

^e^UAI: unprotected anal intercourse.

^f^CAI: condomless anal intercourse.

^g^STD: sexually transmitted disease.

^h^CAS: condom anal sex.

^i^PrEP: pre-exposure prophylaxis.

^j^IG: intervention group.

^k^LGBTQ: lesbian, gay, bisexual, transgender, and queer.

For the modality type of the 34 digital interventions, 20 (59%) were web-based interventions (eg, conducted through interactive websites [53,54,56,57,59,61,64-68,72,78,81,83,85] or multimedia software [75,76,82]); 7 (21%) were mobile-based interventions (eg, through mobile apps [62,63,70,74,79] or SMS text messaging [55,60]); 5 (15%) were video-related (eg, video games [69,73,80], educational videos [58], and videoconference [71]); 2 (6%) interventions were conducted through social media [52,77], and 1 (3%) through a combination of videoconference, SMS text messaging, and social media [84].

In total, 9 (27%) of the 34 interventions were delivered at in-person sessions under the supervision of research staff [53,54,62,73,75,76,79,80,82], while the remaining interventions (n=25, 73%) were operated and delivered remotely. All 3 interventions using multimedia software required on-site delivery with a relatively small sample size (n=56 to 178) [75,76,82], hinting the limitation of such modality type in large-scale community implementation.

### Theories and BCTs

Table 4 provides an overview of theories and BCTs used in all studies. Of the 34 studies, 23 (68%) interventions were theory-based. Cognitive-based theories, including Integrated Behavioral Model [67,68], Information- Motivation- Behavior Skills Model [65,66], Social Cognitive Theory [69,73,82,84], behavioral theory on Dyadic Health model [71], as well as emotion-based approaches, such as Rational Emotive Behavior Therapy theory [55] and Psychological Wise theory [83], were used in these studies. In addition, over half of studies (21/34, 62%) included the BCTs across the 3 hierarchical levels under the Transtheoretical Model (Figure 1), and the other 13 studies included BCTs solely at level 1 and level 2.

**Table 4:** Theoretical frameworks and behavior change techniques (BCTs) used in included interventions (N=34).

Author (year)	Theory used	BCTs used^a^
Anand et al [59], (2020)	—^b^	Level 1: 1, 2, 3, 6, 8, 9, 13 Level 2: 4, 17, 20 Level 3: 18, 23
Bauermeister et al [70], (2019)	Cognitive- emotional decision-making framework	Level 1: 1, 2, 6, 8 Level 2: 4, 5, 17, 20 Level 3: 12
Brody et al [60], (2022)	—	Level 1: 1, 2, 8 Level 2: 4, 20
Bull et al [78], (2009)	Theory of reasoned action and planned behavior and social cognitive theory	Level 1: 1, 2, 3, 6, 19 Level 2: 4 Level 3: 18
Bull et al [77], (2012)	—	Level 1: 1, 3, 6, 8, 19 Level 2: 4, 5, 20
Christensen et al [69], (2013)	Theory of planned behavior and social cognitive theory	Level 1: 1, 2, 6, 8, 13 Level 2: 4, 5, 7, 17, 22 Level 3: 24
Cordova et al [79], (2020)	Ecodevelopment theory and empowerment theory	Level 1: 1, 2, 6, 8 Level 2: 4, 20
Ezegbe et al [52], (2018)	Rational emotive behavior therapy theory	Level 1: 1, 2, 6, 19 Level 2: 4, 17, 21, 22
Fiellin et al [80], (2017)	—	Level 1: 1, 2, 3, 6, 8, 19 Level 2: 4, 5, 17
Hightow-Weidman et al [68], (2012)	Integrated behavioral model	Level 1: 1, 2, 3, 6, 8, 13, 19 Level 2: 4, 5, 7, 20 Level 3: 12
Hightow-Weidman et al [67], (2019)	Integrated behavioral model	Level 1: 1, 2, 3, 6, 8, 13, 19 Level 2: 4, 5, 7, 20, 21 Level 3: 12, 18
Jones et al [81], (2013)	Sex script theory and theory of power as knowing participation in change	Level 1: 6, 8, 19 Level 2: 4, 5, 22
Klein and Card [82], (2011)	Social cognitive theory and the theory of gender and power	Level 1: 1, 2, 3, 6, 8, 9, 13, 19 Level 2: 4, 5, 20
Lau et al [56], (2016)	—	Level 1: 1, 2, 3, 6, 8 Level 2: 4, 20
Levy et al [61], (2021)	Cognitive dissonance	Level 1: 1, 13 Level 2: 4, 7
Logie et al [55], (2023)	—	Level 1: 1, 2, 8, 9 Level 2: 4, 5, 17, 20 Level 3: 18
Marsch et al, [75], (2011)	—	Level 1: 1, 2, 6, 8 Level 2: 4, 5, 20
Marsch et al [76], (2015)	—	Level 1: 1, 2, 6, 8 Level 2: 4, 5, 20
McCrimmon et al [83], (2024)	Psychological wise theory	Level 1: 1, 2, 3, 6, 8, 9, 13, 19 Level 2: 4, 5, 10, 17, 22 Level 3: 12, 25
Mustanski et al [66], (2013)	Information-motivation-behavior-skills model	Level 1: 1, 2, 3, 6, 8, 9, 13, 19 Level 2: 4, 5, 10, 17 Level 3: 11, 18, 23
Mustanski et al [65], (2018)	Information-motivation-behavior-skills model	Level 1: 1, 2, 3, 6, 8, 9, 13, 19 Level 2: 4, 5, 10, 17 Level 3: 11, 18, 23
Nelson et al [64], (2022)	—	Level 1: 1, 2, 6, 8, 9, 13, 19 Level 2: 4, 5, 7
Newcomb et al [71], (2023)	Dyadic health model	Level 1: 1, 2, 3, 6, 8, 9, 13, 19 Level 2: 4, 5, 16, 17, 20, 21 Level 3: 18, 23, 24, 25
Peskin et al [73], (2015)	Social cognitive behavioral theory	Level 1: 1, 2, 3, 6, 8, 19 Level 2: 4, 5, 17, 22 Level 3: 11, 12
Santa Maria et al [74], (2021)	Information-motivation-behavior model	Level 1: 1, 2, 6, 8, 13 Level 2: 4, 17 Level 3: 11, 23
Schnall et al [63], (2022)	Social learning theory	Level 1: 1, 2, 3, 6, 8, 19 Level 2: 4, 5, 22 Level 3: 11, 18, 25
Swendeman et al [84], (2024)	Social cognitive theory	Level 1: 1, 2, 3, 6, 13, 14, 19 Level 2: 4, 5, 7, 10, 17, 20, 21 Level 3: 11, 18, 25
Widman et al [72], (2018)	Reasoned action model and fuzzy trace theory	Level 1: 1, 2, 3, 6, 8, 9, 13, 19 Level 2: 4, 5, 17 Level 3: 12, 25
Widman et al [85], (2020)	Reasoned action model and fuzzy trace theory	Level 1: 1, 2, 3, 6, 8, 9, 13, 19 Level 2: 4, 5, 17 Level 3: 12, 25
Wray et al [62], (2019)	—	Level 1: 1, 2, 6, 13, 19 Level 2: 4, 5, 10, 17, 20, 22 Level 3: 11, 12, 25
Ybarra et al [53], (2013)	Information-motivation-behavior model	Level 1: 1, 2, 3, 6, 8, 9 Level 2: 4 Level 3: 18
Ybarra et al [54], (2015)	Information-motivation-behavior model	Level 1: 1, 2, 3, 6, 8, 9 Level 2: 4 Level 3: 18
Yi et al [57], (2024)	Cognitive behavioral therapy	Level 1: 1, 2, 8, 9, 13 Level 2: 4, 5, 10, 17, 20, 22 Level 3: 11, 12, 18, 24
Zhang et al [58], (2024)	—	Level 1: 1, 2, 3, 8, 9 Level 2: 4, 20

^a^Level 1 to level 3 are 3 hierarchical levels of BCTs based on BCT taxonomy developed by Abraham and Michie [29]: level 1, to provide information and resources; level 2, to initiate action; level 3, to manage and maintain health behaviors for a sustainable change.

^b^Not available.

The frequency of each BCT used in all studies is shown in Table 5. The most commonly used BCTs were prompt intention formation (34/34, 100%), provide information about behavior-health link (33/34, 97%), provide information on consequences (31/34, 91%), provide instruction (30/34, 88%), provide general encouragement (29/34, 85%), prompt barrier identification (23/34, 68%), provide information about others’ approval (20/34, 59%), and provide opportunities for social comparison (19/34, 56%). Unused BCTs include provide contingent rewards, teach to use prompts or cues, and time management. The proportion of BCTs used under each level was 67%, 40%, and 17% for level 1, level 2, and level 3, respectively. All interventions included BCTs from level 1 and level 2. However, BCTs from level 3 were more frequently used in studies targeting older adolescents (aged ≥18 years) [55,57,62,65-71,74,84] compared to younger adolescents (aged <18 years) [53,54,63,72,73,83,85]. Specifically, level 3 BCTs in studies targeting younger adolescents included follow-up prompts [53-55,57,63,71,84], prompt self-monitoring of behavior [57,72,73,83,85], motivational interviewing [63,71,72,83-85], and prompt review of behavioral goals [57,63,73,84], while those targeting older adolescents included prompt review of behavioral goals [62,65,66,74], prompt self-monitoring of behavior [62,67,68,70], relapse prevention [65,66,71,74], stress management [57,69,71], follow-up prompts [65-67], and motivational interviewing [62,80].

**Table 5:** Frequencies of behavior change techniques (BCTs) used across all studies and studies with different improved outcomes.

BCT number	BCT	All studies (N=34), n (%)	Studies with improved HIV-related knowledge (n=11), n (%)	Studies with improved condom-use self-efficacy (n=7), n (%)	Studies with improved condom use (n=14), n (%)	Combined average percentage of improved outcomes (%)
4	Prompt intention formation	34 (100)	11 (100)	7 (100)	14 (100)	100
1	Provide information about behavior-health link	33 (97)	11 (100)	7 (100)	13 (93)	97
2	Provide information on consequences	33 (97)	11 (100)	7 (100)	13 (93)	97
8	Provide instruction	33 (97)	10 (91)	7 (100)	14 (100)	97
6	Provide general encouragement	30 (88)	10 (91)	6 (86)	13 (93)	91
5	Prompt barrier identification	27 (79)	8 (73)	7 (10)	11 (79)	81
3	Provide information about others’ approval	23 (68)	7 (64)	4 (57)	11 (79)	69
19	Provide opportunities for social comparison	21 (62)	7 (64)	4 (57)	9 (64)	63
20	Plan social support or social change	20 (59)	5 (45)	4 (57)	9 (64)	56
17	Prompt practice	19 (56)	7 (64)	5 (71)	6 (43)	56
13	Provide feedback on performance	16 (47)	4 (36)	3 (43)	8 (57)	48
9	Model or demonstrate the behavior	15 (44)	6 (55)	4 (57)	6 (43)	50
18	Use follow-up prompts	12 (35)	3 (27)	1 (14)	5 (36)	28
12	Prompt self-monitoring of behavior	11 (32)	3 (27)	4 (57)	3 (21)	40
11	Prompt review of behavioral goals	8 (24)	1 (9)	1 (14)	3 (21)	15
22	Prompt self-talk	8 (24)	3 (27)	1 (14)	3 (21)	22
25	Motivational interviewing	8 (24)	3 (27)	2 (29)	2 (14)	22
7	Set graded tasks	6 (18)	0 (0)	0 (0)	3 (21)	9
10	Prompt specific goal setting	6 (18)	1 (9)	0 (0)	2 (17)	9
23	Relapse prevention	5 (15)	0 (0)	0 (0)	4 (29)	12
21	Prompt identification as role model	4 (12)	1 (9)	0 (0)	2 (14)	9
24	Stress management	3 (9)	0 (0)	0 (0)	2 (14)	6
16	Agree behavioral contract	1 (3)	0 (0)	0 (0)	1 (7)	3
14	Provide contingent rewards	0 (0)	0 (0)	0 (0)	0 (0)	0
15	Teach to use prompts or cues	0 (0)	0 (0)	0 (0)	0 (0)	0
26	Time management	0 (0)	0 (0)	0 (0)	0 (0)	0

### Results and BCTs per Improved Outcome

Three key outcome measurements were identified among the included 34 studies, including (1) HIV knowledge (n=16, 47%), (2) condom-use self-efficacy (n=15, 44%), and (3) frequency of condom use (n=26, 76%). Over half (20/34, 59%) of the interventions had an intervention completion rate higher than or equal to 85%. It is estimated that the high completion rate might be a result of school-based and clinic-based recruitment. Follow-up measurements were commonly carried out immediately after intervention, at 3 and 6-month follow-ups. Variations existed depending on the nature and duration of studies (Tables 2 and 3).

In total, 11 (69%) of the 16 studies that included HIV knowledge as an outcome reported a substantial improvement in HIV knowledge. In addition, 7 (47%) of 15 studies that included condom-use self-efficacy as an outcome reported a statistically significant increase in condom-use self-efficacy. Moreover, 14 (54%) of 26 studies that included condom use as an outcome reported a statistically significant increase in condom use frequency. Table 5 summarizes the frequencies of BCTs linked to each outcome indicators in studies with significant improvements. Prompt intention formation (100%), provide information about behavioral-health link (97%), provide information on consequences (97%), provide instruction (97%), and provide general encouragement (91%) were the most commonly used BCTs among all the 3 improved target outcomes. Prompt practice (5/7, 71%) and prompt self-monitoring of behavior (4/7, 57%) were more frequently used in studies to drive improvement in condom-use self-efficacy compared to other outcome indicators, while a more diverse and even use of the 26 BCTs across the 3 levels was observed in studies with an increase in condom use frequency.

### Quality Assessment

The quality assessment of included studies based on the Joanna Briggs Institute tool is provided in Table 6. All 34 studies included were randomized. Among these, 10 (29%) studies reported the use of concealed allocation [52,57-59,63,65,66,71,81,84], and 31 (91%) studies demonstrated baseline similarity between intervention and control groups [52-54,56-77,79-83,85]. Blinding was less frequently reported, with 5 (15%) studies documenting participant blinding [52,63,65,66,84], 5 (15%) studies documenting outcome assessor blinding [52,55,56,60,82], and 7 (21%) studies documenting blinding of deliverers [60,63,65,66,72,81,84]. Most studies (31/34, 91%) ensured that the groups were identical except for the intervention [52-54,56,57,59-70,72-85]. All studies reported identical outcome measurements, and 32 (94%) studies used reliable outcome measurements, with 2 (6%) studies lacking this information [55,60]. Follow-up analysis was conducted in 23 (68%) studies [52-54,56,59,60,62,65,67,69,70,72-81,84,86], and 31 (91%) studies performed analysis according to correct groupings [52-56,58-71,73-82,84,85]. The quality score of the included studies ranged from 45% [55,83] to 91% [52,65], with 24 (71%) studies receiving the score <70%, and 10 (29%) studies receiving a quality score >70%.

**Table 6:** Risk of bias assessment for included studies using the Joanna Briggs Institute appraisal tool (N=34).

Author (year)	Quality (%)	Randomized	Concealed allocation	Similar to baseline	Participants blind to assignment	Deliverers blind to assignment	Identical groups except intervention of interest	Outcome assessors blind to assignment	Identical outcome measurement	Reliable outcome measurement	Analysis of follow-up	Analysis according to correct groupings
Anand et al [59], (2020)	73	Yes	Yes	Yes	No	No	Yes	Unclear	Yes	Yes	Yes	Yes
Bauermeister et al [70], (2019)	64	Yes	Unclear	Yes	Unclear	Unclear	Yes	Unclear	Yes	Yes	Yes	Yes
Brody et al [60], (2022)	73	Yes	Unclear	Yes	No	Yes	Yes	Yes	Yes	No	Yes	Yes
Bull et al [78], (2009)	55	Yes	Unclear	No	Unclear	Unclear	Yes	Unclear	Yes	Yes	Yes	Yes
Bull et al [77], (2012)	64	Yes	Unclear	Yes	Unclear	Unclear	Yes	Unclear	Yes	Yes	Yes	Yes
Christensen et al. [69], (2013)	64	Yes	No	Yes	Unclear	Unclear	Yes	Unclear	Yes	Yes	Yes	Yes
Cordova et al [79], (2020)	64	Yes	Unclear	Yes	Unclear	No	Yes	Unclear	Yes	Yes	Yes	Yes
Ezegbe et al [52], (2018)	91	Yes	Yes	Yes	Yes	No	Yes	Yes	Yes	Yes	Yes	Yes
Fiellin et al [80], (2017)	64	Yes	Unclear	Yes	Unclear	Unclear	Yes	Unclear	Yes	Yes	Yes	Yes
Hightow-Weidman et al [68], (2012)	55	Yes	Unclear	Yes	Unclear	Unclear	Yes	Unclear	Yes	Yes	No	Yes
Hightow-Weidman et al [67], (2019)	64	Yes	Unclear	Yes	Unclear	Unclear	Yes	Unclear	Yes	Yes	Yes	Yes
Jones et al [81], (2013)	82	Yes	Yes	Yes	Unclear	Yes	Yes	Unclear	Yes	Yes	Yes	Yes
Klein and Card [82], (2011)	64	Yes	Unclear	Yes	Unclear	No	Yes	Yes	Yes	Yes	No	Yes
Lau et al [56], (2016)	73	Yes	No	Yes	No	No	Yes	Yes	Yes	Yes	Yes	Yes
Levy et al [61], (2021)	55	Yes	Unclear	Yes	Unclear	Unclear	Yes	Unclear	Yes	Yes	No	Yes
Logie et al [55], (2023)	45	Yes	No	No	Unclear	Unclear	No	Yes	Yes	No	Yes	Yes
Marsch et al [75] (2011)	64	Yes	Unclear	Yes	Unclear	Unclear	Yes	Unclear	Yes	Yes	Yes	Yes
Marsch et al [76], (2015)	64	Yes	Unclear	Yes	Unclear	Unclear	Yes	Unclear	Yes	Yes	Yes	Yes
McCrimmon et al [83], (2024)	45	Yes	Unclear	Yes	Unclear	Unclear	Yes	Unclear	Yes	Yes	No	No
Mustanski et al [66], (2013)	82	Yes	Yes	Yes	Yes	Yes	Yes	Unclear	Yes	Yes	No	Yes
Mustanski et al [65], (2018)	91	Yes	Yes	Yes	Yes	Yes	Yes	Unclear	Yes	Yes	Yes	Yes
Nelson et al [64], (2022)	55	Yes	Unclear	Yes	Unclear	Unclear	Yes	Unclear	Yes	Yes	No	Yes
Newcomb et al [71], (2023)	55	Yes	Yes	Yes	Unclear	Unclear	No	Unclear	Yes	Yes	No	Yes
Peskin et al [73], (2015)	64	Yes	Unclear	Yes	Unclear	No	Yes	Unclear	Yes	Yes	Yes	Yes
Santa Maria et al [74], (2021)	55	Yes	Unclear	Yes	Unclear	Unclear	Yes	Unclear	Yes	Yes	Yes	Yes
Schnall et al [63], (2022)	82	Yes	Yes	Yes	Yes	Yes	Yes	Unclear	Yes	Yes	No	Yes
Swendeman et al [84], (2024)	82	Yes	Yes	No	Yes	Yes	Yes	Unclear	Yes	Yes	Yes	Yes
Widman et al [72], (2018)	73	Yes	Unclear	Yes	Unclear	Yes	Yes	Unclear	Yes	Yes	Yes	Yes
Widman et al [85], (2020)	64	Yes	Unclear	Yes	Unclear	Unclear	Yes	Unclear	Yes	Yes	Yes	Yes
Wray et al [62], (2019)	64	Yes	No	Yes	No	No	Yes	No	Yes	Yes	Yes	Yes
Ybarra et al [53], (2013)	64	Yes	No	Yes	No	No	Yes	No	Yes	Yes	Yes	Yes
Ybarra et al [54], (2015)	64	Yes	No	Yes	No	No	Yes	No	Yes	Yes	Yes	Yes
Yi et al [57], (2024)	55	Yes	Yes	Yes	No	No	Yes	Unclear	Yes	Yes	No	No
Zhang et al [58], (2024)	64	Yes	Yes	Yes	No	No	Yes	Unclear	Yes	Yes	No	Yes

## Discussion

### Use of BCTs and Theories

From our understanding, this study is the first review on the use of BCTs in digital HIV interventions among adolescents and young people. The findings showed that among the 3 levels of BCTs, BCTs at level 1 are the most frequently used with an average frequency of 67%. BCTs at level 1 help to deepen participants’ understanding of HIV and the role of condoms in preventing sexually transmitted diseases. By enhancing and consolidating important knowledge, higher motivation will be generated to encourage change. This is especially useful for people at the precontemplation and contemplation stages. In contrast, level 2 BCTs mark the starting point of turning internal ideas into actual behavioral change, or in other words mobilizing participants to enter the preparation and action stage from precontemplation stage, according to the transtheoretical model. Level 2 BCTs had an average frequency of 40% in this study, which is less commonly used among the 34 studies compared to level 1 BCTs. Among all, level 3 BCTs were at the highest hierarchical level as they are the most complicated to implement though potentially most impactful in causing long-term change. Level 3 BCTs were the least used among all 3 levels with an average frequency of only 17%. The use of BCTs from 3 hierarchical levels in this systematic review matches with the rule of thumb for at-risk populations under the transtheoretical model. Specifically, level 1 BCTs were commonly used for the largest group of recipients from precontemplation (40%) and contemplation (40%) stage, while level 2 BCTs were less frequently used for populations at the preparation stage (20%) [32]. It is also important to note that the majority of the included studies (23/34, 68%) were observed to be supported by or constructed based on behavior change models or theories. No substantial difference was observed in the number of BCTs used under each theory in this review. In other words, the impact of theories on the pattern of BCTs used seems to be minimal.

### BCTs Linked to Improved Outcomes

Findings of the study found that level 1 BCTs with a provision nature were the most commonly used in bringing significant improvement in HIV knowledge, indicating that *providing* information and instructions can be an effective method to increase knowledge of participants. Some level 2 BCTs were used for consolidating information, such as prompt practice. Level 3 BCTs were rarely used regarding this outcome, indicating that higher level BCTs concerning larger degrees of self-initiated participation were not considered necessary in enhancing HIV knowledge. In contrast, a higher percentage use of level 2 BCTs was observed in studies with significant increase in condom-use self-efficacy. As suggested by the self-efficacy theory [89,90], self-efficacy can be determined by past experience (performance accomplishment), modeling by others (vicarious experience), coaching and evaluative feedback (social persuasion), as well as physiological and emotional states. Level 2 BCTs, such as prompt practice, plan social support, and motivational interviewing, were more frequently used together with level 1 BCTs, including providing feedback on performance and model the behavior, to provide and initiate experience, and modeling and coaching feedback to participants to effectively enhance one’s condom-use self-efficacy.

Finally, studies with a significant increase in condom use frequencies used level 3 BCTs the most among all 3 outcome indicators, suggesting the importance of managing an action for a sustained behavioral change. Involved level 3 BCTs include use follow-up prompts, relapse prevention, and stress management, which are rarely or have never been used in achieving the other 2 outcome indicators. In addition, provide information about others’ approval, provide feedback on performance (level 1), and plan social support (level 2) were used more frequently in increasing condom use, showing the importance of perceived social norms on driving actual behavior change. Findings of this paper match with those from relevant systematic reviews of digital health interventions of other health conditions, which show that provide encouragement, provide information about behavior-health links, provide instructions, and provide information on consequences were the most frequently used BCTs [91,92].

### Advantages of Digital Platforms for Delivering BCTs in HIV Prevention

Digital platforms offer unique features that allow the application of certain BCTs in HIV prevention interventions. Web-based interventions can reach a wider audience, including hard-to-reach populations (eg, MSM), and offer cost-effective solutions compared to traditional face-to-face interventions [93]. In addition, the interactive and engaging nature of digital platforms helps to deliver BCTs more effectively, making them a powerful tool in HIV prevention efforts. For instance, customizable avatars allow participants to choose characters that share their race and cultural background, which makes it easier for participants to empathize and imagine the cases in reality. This customizable function facilitates the use of BCTs, such as prompt barrier identification and providing opportunities for social comparisons. Anonymous interactions on forums and video-uploading platforms enable participants to express genuine concerns and thoughts freely. These interactions incorporate BCTs like providing information about others’ approval and facilitating planning of social change [94]. Such anonymity ensures that the youth feel safe and supported, which is particularly important for sensitive topics like HIV prevention. Tailor-made content based on risk assessment results and preset goals ensures that messages are accurate and relatable to targeted populations, which can enhance the relevance and impact of the interventions. The process of information through the central route is deepened with the use of follow-up prompts and providing feedback on performance [95], ensuring that participants fully understand and engage with the information provided. Finally, unlocking graded modules, for example, quizzes or chapters with elevating difficulty, helps participants gradually build a solid foundation of knowledge and behavioral change. BCTs like setting graded tasks and providing performance feedback are effectively integrated into digital interventions, ensuring continuous engagement and learning in HIV prevention.

### Public Health Contribution and Implications

The youth demographic is particularly vulnerable due to the high level of engagement in risky sex and barriers to accessing traditional health care services. The focus on digital interventions is significant in this underserved and hard-to-reach population. The internet offers a wider yet more complex platform for health education. Its distinctive attributes, such as rapidly evolving trends, a multitude of information sources, and diverse user demographics, can make it challenging to effectively use digital health interventions. To navigate this evolving landscape of health education, it is beneficial to examine successful strategies that incorporate commonly used BCTs in digital interventions.

The findings of this review offer valuable guidance and insights for the design and implementation of future HIV prevention programs aimed at adolescents and young people. The BCTs identified in this review serve as a valuable reference for further studies, enabling researchers to select appropriate BCTs for intervention development and improvement. The most used BCTs identified in this review included prompting intention formation, providing information about behavior-health links, providing information on consequences, providing instructions, and providing general encouragement, which is important in fostering awareness and motivation for behavior change. Interventions showing improvements in HIV knowledge mostly included BCTs with a provision focus, such as providing information on consequences, behavior-health links, and encouragement. This suggests that educational components are essential for building foundational knowledge and promoting initial engagement and readiness for behavior change. In this review, BCTs aimed at initiating actions (eg, prompts for intention formation and barrier identification) were commonly observed in studies reporting a significant increase in condom-use self-efficacy, which provides evidence for bridging the gap between awareness and action. While to improve actual condom use, BCTs focused on managing and sustaining behavior (eg, use of follow-up prompts and relapse prevention) may need to be incorporated into the interventions. Considering the diverse range of outcomes to be achieved, hierarchical levels of BCTs should also be taken into account to design tailored and effective interventions. It is recommended that public health practitioners use behavior change theories as a foundation, using BCTs at different levels to select intervention activities and deliver pertinent messages for maximizing program effectiveness.

In addition, future studies can delve into identifying intrinsic and extrinsic motivational techniques within the current BCT taxonomy. Exploring the effectiveness of emotive techniques in driving behavior change in digital interventions could be another promising avenue for investigation. Moreover, the digital nature of the included interventions may allow for the scalability and adaptability of these findings to other topics beyond HIV prevention, particularly those that share similar characteristics. By leveraging the knowledge gained from this review, we can enhance the effectiveness of HIV prevention programs for adolescents and young people and facilitate border implementation.

### Limitations

There were some limitations of the study. First, some BCTs may not be easily observable and thus might have limited the identification of the exact BCTs used. Four BCTs, namely provide contingent rewards, teach to use prompts or cues, agree behavioral contract, and time management were never used in the included studies. Among these, teach to use prompts or cues and time management can be unobservable techniques that are hidden in message contents, rather than being described as intervention activities. The limited description of intervention messages can conceal certain BCTs, leading to bias in analysis and estimation of BCT effects. Also, definitions of some BCTs, such as provide general encouragement and prompt intention formation, may be too general and ambiguous for having a meaningful analysis. Some BCTs, such as prompt intention formation (34/34, 100%), provide information about behavioral-health link (33/34, 97), provide information on consequences (33/34, 97%), and provide instruction (33/34, 97%), were used in almost every study. Their high frequency can be due to their broad definitions, making them almost necessary for all behavioral change interventions.

Second, emotive techniques are proven to be highly effective in affecting one’s judgment and decision-making [96] but are not included in the 26-item taxonomy of BCTs. Fear appeal is an emotive technique used in the intervention of one of the included studies but failed to be categorized into any of the BCTs. The omission of important emotion-driven behavior change approaches in the BCTs may have limited the applicability of the taxonomy in other settings.

Third, counting the frequencies of BCTs used may not fully represent the associations between BCTs and significant improvements. Effect size of individual BCT is difficult to ascertain as multiple BCTs are used in one study. Due to the lack of effect size calculation, it is hard to determine whether the high frequency of use of a BCT is due to its effectiveness or its simplicity in application. It also limited meta-analytic evaluations. Hence, it is difficult to fully attribute the significant improvements in results to the use of a specific BCT.

Finally, this review included study samples aged 10 to 30 years, consisting of early adolescents, middle adolescents, and late adolescents or young people. The age difference of targeted populations may influence the effectiveness of the BCTs provided, as the risk levels of HIV infection and challenges in enacting certain BCTs may differ among early adolescents and young people. Future studies can consider age-specific tailoring to address the potential differences.

### Conclusions

This systematic review has identified the most commonly used BCTs used in digital HIV prevention programs targeting adolescents and young people. The findings can serve as important references for future interventions to provide more effective approaches for HIV prevention on the internet.

## Appendix

Multimedia Appendix 1 Search strategy.

Multimedia Appendix 2 PRISMA (Preferred Reporting Items for Systematic Reviews and Meta-Analyses) checklist.

Multimedia Appendix 3 Eligibility criteria.

## Abbreviations

BCT: behavior change technique
MSM: men who have sex with men
RCT: randomized controlled trial

## Data Availability

Data sharing is not applicable to this paper as no datasets were generated or analyzed during this study.
