# Supplementary material for: Use of Behavior Change Techniques in Digital HIV Prevention Programs for Adolescents and Young People: Systematic Review
Source: JMIR Public Health Surveill. 2025 Apr 28;11:e59519. doi: 10.2196/59519 (PMC12070010; doi:10.2196/59519)
Supplement: Multimedia Appendix 1 [file publichealth_v11i1e59519_app1.docx]

### **Appendix I. Literature Search Strategy**

This review conducted literature search across four databases: PubMed, EMBASE, Cochrane Library, and APA PsycINFO. Search terms were developed in five domains—setting, intervention, outcome, population, and study design—using Boolean operators to link terms within and across these categories.

**Search Terms and Boolean Strategy**

The search terms in the five domains included the following: 1) Setting: Internet-related (e.g., online, web, Internet), 2) Intervention: HIV prevention, 3) Outcome: HIV knowledge or condom use, 4) Population: youth-related (e.g., adolescent, young), and 5) Study design: Randomized controlled trial or trial. The search terms across different domains were linked using Boolean logic, as below.

| Database | Search Query |
| --- | --- |
| PubMed | ((((("Online" OR "Web" OR "Internet" OR "Computer" OR "Smartphone" OR "mHealth" OR "eHealth")) AND ("HIV prevention" OR "HIV intervention" OR "AIDS prevention" OR "HIV risk reduction")) AND ("Sex education" OR "Condom" OR "Unprotected sex" OR "HIV knowledge")) AND ("Adolescent*" OR "Young" OR "Youth" OR "Student*")) AND ("Randomized controlled trial" OR "Controlled clinical trial" OR "RCT" OR "Clinical trial")  LANGUAGE: (English), Peer-reviewed Articles, Timespan=2008-Current (5 Nov 2024), Excluded: Meta-Analysis, Review, Systematic Review |
| Embase and APA PsycInfo | ((online or web or internet or computer or smartphone or mHealth or eHealth) AND (HIV prevention or HIV intervention or AIDS prevention or HIV risk reduction) AND (sex education or condom or unprotected sex or HIV knowledge) AND (adolescent* or young or youth or student*) AND (randomized controlled trial or controlled clinical trial or RCT or clinical trial)).mp.limit to English language, yr= “2008-Current (5 Nov 2024)” |
| Cochrane library | ((((((online) OR (web)) OR (internet)) OR (computer)) OR (smartphone)) OR (mHealth)) OR (eHealth) in All Text AND (((HIV prevention) OR (HIV intervention)) OR (AIDS prevention)) OR (HIV risk reduction) in All Text AND (((sex education) OR (condom)) OR (unprotected sex)) OR (HIV knowledge) in All Text AND (((adolescent*) OR (young)) OR (youth)) OR (student*) in All Text AND (((randomized controlled trial) OR (controlled clinical trial)) OR (RCT)) OR (clinical trial) in Title Abstract Keyword - with Cochrane Library publication date from Jan 2008 to present (5 Nov 2024), in Trials (Word variations have been searched) |
