# Supplementary material for: Use of Behavior Change Techniques in Digital HIV Prevention Programs for Adolescents and Young People: Systematic Review
Source: JMIR Public Health Surveill. 2025 Apr 28;11:e59519. doi: 10.2196/59519 (PMC12070010; doi:10.2196/59519)
Supplement: Multimedia Appendix 3 [file publichealth_v11i1e59519_app3.docx]

### **Appendix III. Eligibility criteria for study inclusion**

| **Category** | **Inclusion Criteria** | **Exclusion Criteria** |
| --- | --- | --- |
| **Population** | Adolescents and young people aged 10 to 30 years. Studies with >50% of participants in this age group were included, even if the intervention targeted broader age ranges. This criterion aligns with studies that set 30 years as the cut-off age [49-51]. | Studies that did not focus on this age range or had <50% of participants within this demographic. |
| **Intervention** | 1) Any form of Internet-based intervention, including but not restricted to, websites, health apps, emails, social media, and text messaging;  2) Interventions with HIV prevention components. | 1) Interventions that were not Internet-based;  2) Studies without an explicit HIV prevention component. |
| **Outcome** | HIV prevention outcomes, including improvements in HIV knowledge, condom-use self-efficacy, or increased condom use. | Studies that did not focus on HIV prevention outcomes. |
| **Study Design** | Randomized-controlled trials (RCTs), cluster RCTs, pilot RCTs | Observational, cross-sectional, qualitative, pre/post, case studies, case-control, reviews, meta-analyses |
| **Language** | English | Non-English publications |
| **Published date** | 2008 to Nov 2024 *  *date when last searches were conducted | Before 2008 |
| **Publication type** | Research article reporting findings of eligible study designs | Conference abstracts, protocols, commentaries, uppublished literature, retracted articles |
